# LncRNA TP73‐AS1 promotes nasopharyngeal carcinoma progression through targeting miR-342-3p and M2 polarization via exosomes

**DOI:** 10.1186/s12935-021-02418-5

**Published:** 2022-01-10

**Authors:** Hongchao Yao, Linli Tian, Bingrui Yan, Like Yang, Yushan Li

**Affiliations:** Department of Otolaryngology Head and Neck Surgery, The Second Affiliated Hospital of Harbin Medical University, No. 246 Xue Fu Road, Harbin, 150086 China

**Keywords:** Nasopharyngeal carcinoma, TP73-AS1, miR-342-3p, Exosome, Progression

## Abstract

**Background:**

Nasopharyngeal carcinoma (NPC) is a deadly cancer, mainly presenting in southeast and east Asia. Long noncoding RNAs (lncRNAs) play essential roles in cancer progression. Exosomes are critical for intercellular communication. Thus, the aim of this study was to identify the functional lncRNAs in NPC and its relevant mechanisms.

**Methods:**

Data from public databases were utilized to screen for functional lncRNAs in NPC. Functional and mechanical experiments were performed to determine the role of lncRNAs in NPC and its relative molecular mechanisms. Exosomes derived from NPC cells were isolated to determine their function in tumor-associated macrophages.

**Results:**

LncRNA TP73-AS1 was increased in NPC cells and tissues and was associated with a poor prognosis. TP73-AS1 overexpression promoted proliferation, colony formation, and DNA synthesis of NPC cells while TP73-AS1 knockdown showed opposite roles. TP73-AS1 could directly bind with miR-342-3p. MiR-342-3p overexpression attenuated the effect of TP73-AS1 in NPC cells. Furthermore, TP73-AS1 was transferred by exosomes to promote M2 polarization of macrophages. Lastly, exosomal TP73-AS1 enhanced the motility and tube formation of macrophages.

**Conclusions:**

Together, this study suggests that TP73-AS1 promotes NPC progression through targeting miR-342-3p and exosome-based communication with macrophages and that TP73-AS1 might be an emerging biomarker for NPC.

## Background

Nasopharyngeal carcinoma (NPC) is a deadly cancer taking place in the nasopharynx epithelium, especially in the fossa of Rosenmüller [1]. Additionally, NPC is an uncommon cancer type and the presence of NPC displays distinct geographical distribution such that the majority of new cases (71%) are reported in southeast and east Asia [2]. In China, approximately 42,100 new NPC cases, including 23,320 deaths, were reported in 2013 which accounts for 1.14% of new cancer cases and 0.96% of cancer-associated deaths in that year [2, 3]. Over the past decades, epidemiological evidence suggests that the incidence and mortality of NPC have decreased significantly [4]. However, due to its biological heterogeneity, substantial variations of clinical manifestation of NPC are found in patients with the same clinical stage or similar therapeutic strategies [5]. These observations suggest that the current staging system is inadequate for clinical application for NPC patients. Thus, it is imperative to elucidate the molecular mechanism underlying the progression of NPC.

Long noncoding RNAs (lncRNAs) are a group of noncoding RNAs larger than 200 nt in length [6]. Growing evidence suggests that lncRNAs play essential roles in various biological processes, including stem cell biology [7], embryogenesis [8], and epigenetic regulations [9]. Notably, the critical role of lncRNAs in the development and progression of cancer has caught considerable attention. Numerous studies have demonstrated that lncRNAs are tightly associated with metastasis, migration, invasion, proliferation, as well as apoptosis [10, 11]. For NPC, lncRNA-LINC00460 is upregulated in NPC tissues and promotes tumorigenesis through silencing miR-149-5p [12]. Also, lncRNA PVT1 acts as an oncogenic factor to regulate NPC cell proliferation and radiosensitivity through the involvement of KAT2A acetyltransferase [13]. Moreover, the upregulation of lncRNA ANRIL is associated with NPC cell proliferation, glucose metabolism, and maintaining stem-like properties [14].

Tumor-associated macrophages (TAMs) are the most abundant immune-associated cells in the tumor microenvironment (TME) [15]. It has been reported that circulating macrophages and monocytes are recruited in the TME, thereby facilitating tumor progression [16]. Regarding their function, macrophages exhibit two phenotypes: M1 and M2 [17]. M1 macrophages are responsible for anti-tumor immunity and inflammatory responses, whereas M2 macrophages are involved in wound healing and tumorigenesis [18]. Given the importance of M2 macrophages in TME, the mechanism by which TAMs influence the cellular activities of cancer cells has emerged as a hot topic. Numerous studies have demonstrated that exosomes, the vesicles with 30–150 nm diameter, play an essential role in intercellular communication between TMEs and cancer cells [19–21]. As an essential function, exosomes can transfer bioactive molecules, such as proteins, lipids, RNAs, and noncoding RNAs in intercellular communication, eventually influencing the biological processes of recipient cells [22].

In the present study, we investigated the public NPC dataset of the Cancer Genome Atlas (TCGA) and identified the potential functional lncRNA TP73-AS1 (TP73-AS1) for NPC. Next, we determined the role of TP73-AS1 in the progression of NPC and its related mechanisms in vitro and in vivo. Additionally, we determined that TP73-AS1 functioned as an oncogenic factor binding with miR-342-3p to promote NPC progression. Lastly, we demonstrated that TP73-AS1 could be transferred from NPC to TAMs cells to facilitate the development of TAMs. These results provide a new understanding of the molecular mechanism of the progression of NPC.

## Methods

### Patient samples

Human NPC tissues and adjacent healthy tissues were collected from 12 patients (female: 6; male: 6; age: 47–72 years) in the second Affiliated Hospital of Harbin Medical University from January 2012 to December 2016. Written informed consent was obtained from all patients. The research was approved by the Ethical Committee on Scientific Research of the second Affiliated Hospital of Harbin Medical University. Tissues were collected by fiber optic nasopharyngoscopy from tumor growth sites and the adjacent sites with obvious normal mucosal morphology, respectively. All tissues were stored in − 80 °C until further processing.

### RNA sequencing and 5′- and 3′-rapid amplification of cDNA ends analysis (RACE)

The expression profile of lncRNAs of NPC was obtained from the Gene Expression Omnibus (GEO) dataset GSE95166 using The Cancer Genome Atlas (TCGA) database. Data analysis was carried out using the DEGseq package (R software) as previously described [23]. The threshold for statistically significant difference was log_2_|fold change|≥ 2 and *p* < 0.05. 5′- and 3′-RACE assay were performed using a SMARTer™ RACE cDNA Amplification Kit (Takara Bio, Japan) following the manufacturer’s instructions.

### Cell culture

Human nasopharyngeal carcinoma cell lines (CNE-1, CNE-2, SUNE2, HOEN1, S26) and control human nasopharyngeal epithelial cell line (NP69) were purchased from American Type Culture Collection (ATCC, USA). Cells were cultured in Roswell Park Memorial Institute (RPMI) 1640 medium (Thermo Fisher Scientific, USA) supplemented with 10% fetal bovine serum (FBS; Thermo Fisher Scientific, USA), 100 μg/ml streptomycin, and 100 U/ml penicillin. Human embryonic kidney 293 (HEK293T) cells were cultured with Dulbecco’s modified Eagle’s medium (DMEM; Thermo Fisher Scientific, USA). Human monocytic cell line (THP1) was cultured in RPMI 1640 medium. All cell lines were cultured in a 5% CO_2_ incubator at 37 °C.

### Quantitative real-time PCR

Total RNA of cells, tissues, and exosomes were isolated using TRIzol reagent (Thermo Fisher Scientific, USA) following the manufacturer’s instructions. First-strand cDNA was synthesized using the PrimeScript RT reagent kit (TaKaRa, Japan) following the manufacturer’s instructions. Reverse transcription of miR-342-3p was performed using the PrimeScript miRNA cDNA Synthesis Kit (TaKaRa, Japan). The PCR assay was performed on ABI7900/illumina eco (Applied Biosystems, USA). GAPHD and U6 were used as the internal control for the relative expression of mRNA and miRNA, respectively. Raw data were analyzed using ΔΔCT method [24]. The primer information was as following: GAPDH: 5′-AAGGCTGAGAATGGGAAAC-3′ (Forward) and 5′-TTCAGGGACTTGTCATACTTC-3′ (Reverse), U6: 5′-GCTTCGGCAGCACATATACTAAAAT-3′ (Forward) and 5′-CGCTTCACGAATTTGCGTGTCAT-3′ (Reverse); miRNA-342-3p: 5′-GTGCTATCTGTGATTGAGGGA-3′ (Forward) and 5′-CGGGTGCGATTTCTGTG-3′ (Reverse); TP73-AS1: 5′-CCGGTTTTCCAGTTCT TGCAC-3′ (Forward) and 5′-GCCTCACAGGGAAACTTCATGC-3′ (Reverse) [25].

### Cytoplasmic/nuclear RNA separation assay

Cytoplasmic and nuclear RNA of CNE-2 cells were isolated as previously described [26, 27]. The expression of TP73-AS1 in cellular fractions was determined by quantitative real-time PCR described above.

### Fluorescence in situ hybridization assay

Fluorescence in situ hybridization (FISH) assay was carried out in CNE-2 cells following the manufacturer’s instructions. Cy3-labeled TP73-AS1 was obtained from GenePharma (Shanghai, China). DAPI was used to label the nuclei. Red PKH26 dye (GenePharma, China) was used to label exosomes. The images were taken under a fluorescence microscope (Nikon, Japan).

### Cell transfection

TP73-AS1 cDNA was cloned into pcDNA3.1 plasmid following the manufacturer’s instructions (Thermo Fisher Scientific, USA). Short interference RNAs against TP73-AS1 were obtained from GenePharma (Shanghai, China). MiRNA-342-3p mimic, inhibitor, and corresponding negative controls were purchased from GenePharma (Shanghai, China). Cell transfection was performed using the Lipofectamine™ 3000 (Invitrogen, USA) according to the manufacturer’s instructions.

### Xenograft model

Animal experiments were carried out according to the Regulations for the Management of Laboratory Animals published by the Ministry of Science and Technology of the People's Republic of China and the Guidelines for Proper Conduct of Animal Experiments of Harbin Medical University. Animal experiment protocols were approved by the Institutional Animal Care and Use Committee of the second Affiliated Hospital of Harbin Medical University (AH-2013-483A). CNE-2 cells (1 × 10^6^) transfected with the pcDNA3.1 plasmid overexpressing TP73-AS1 or the negative control were subcutaneously injected into the flank of C57BL/6 male mice (6–8 weeks old). After 5 weeks, mice were sacrificed. The weight and volume of tumors were quantified. Next, the tumor tissues were collected to perform hematoxylin and eosin (H&E) staining for morphology evaluation.

### Immunohistochemical assay

Immunohistochemical assay was performed for detecting Ki67 expression in tumor tissues as previously described [26]. An antibody against Ki67 (Abcam, USA) was used to stain Ki67 according to the manufacturer’s instructions. Ki67-positive cells were counted using Imagej software [28].

### Luciferase reporter assay

The wild-type or mutant TP73-AS1 was cloned into the pmirGLO vector. HEK293T cells were seeded on a 6-well plate and transfected with modified pmirGLO vectors plus miR-342-3p mimic using the Lipofectamine™ 3000 (Invitrogen, USA) according to the manufacturer’s instructions. Luciferase reporter assay was performed using the Dual-Luciferase Reporter Assay System (Promega, USA) according to the manufacturer’s instructions.

### RNA immunoprecipitation assay

RNA immunoprecipitation (RIP) assay was performed using the Magna RIP™ RNA-Binding Protein Immunoprecipitation Kit (Millipore, USA) according to the manufacturer’s instructions. Antibodies against AGO2 and IgG were used in the RIP assay according to the manufacturer’s instructions. Precipitated RNA–protein complex was used to synthesize cDNA.

### MTT assay

In 6-well plates, 1 × 10^4^ cells were seeded. Next, 20 μl of 5 mg/ml MTT was added to each well and incubated for 4 h. After carefully removing the supernatants, 100 μl DMSO was added to each well. Cell proliferation was determined using a microplate reader at 570 nm (Bio-Rad, USA).

### Colony formation, migration, and invasion assay

After corresponding treatments, CNE-2 cells (1 × 10^3^) were seeded in 6-well plates. After 14 days, cell colonies were fixed with ethanol and stained with crystal violet for 20 min. The colonies were photographed and counted at five random sites. Migration and invasion assays were performed in CNE-2 cells (1 × 10^5^) using Cell Migration & Invasion Kits (Sigma-Aldrich, USA) according to the manufacturer’s instructions.

### Tube formation assay

Tube formation assay was performed as previously described [29]. Briefly, HUVECs were suspended in the conditioned medium and seeded onto the gel. After the 6 h incubation, images were taken under a bright-field microscope. The total length of tubes in each image were quantified using ImageJ software [28].

### EdU and apoptosis assay

After corresponding treatments, CNE-2 cells (1 × 10^4^) were seeded into 6-well plates. The EdU assay was performed using the Click-iT™ EdU Cell Proliferation Kit (Thermo Fisher Scientific, USA) according to the manufacturer’s instructions. Images were taken under a fluorescence microscope (Nikon, Japan). Cell apoptosis was determined using the Annexin-V-FLUOS Staining Kit (Sigma-Aldrich, USA) according to the manufacturer’s instructions.

### Exosome isolation

Exosomes were isolated from CNE-2 cells transfected with the pcDNA3.1 plasmid overexpressing TP73-AS1 or the negative control through a hyper-centrifugation protocol as previously described [30, 31]. The morphology of exosomes was determined via transmission electron microscopy (TEM; Philips, USA) as previously described [32]. The size distribution of exosomes was determined via nanoparticle tracking analysis (NTA) as previously described [33]. Exosomal protein was determined using the Pierce BCA Protein Assay Kit (Thermo Fisher Scientific, USA) according to the manufacturer’s instructions.

### Western blotting assay

Western blotting assay was performed as previously described [34]. Briefly, total protein of exosomes was isolated using the cell lysis buffer (Beyotime Institute of Biotechnology, China). Total protein was then separated by 10% SDS-PAGE gels and transferred to PVDF membranes (Millipore, USA). The membranes were blocked with 5% skim milk and incubated with antibodies against CD63 (1:1000), Alix (1:1000), and Hsp70 (1;500) (Abcam, USA) for 16 h at 4 °C. Next, the membranes were incubated with their corresponding secondary antibodies. Optical density of protein bands was detected using the Uvitec Alliance software (Eppendorf, Germany).

### Statistical analysis

Data were expressed as the mean ± SD. Each experimental group included at least three replicates. Statistical analysis was performed using GraphPad Prism Software 8 (GraphPad Software, USA). Mean differences between groups were analyzed using the Student’s t-test. *P* < 0.05 was considered as statistically significant.

## Results

### TP73-AS1 is involved in the progression of NPC

**Fig. 1:**
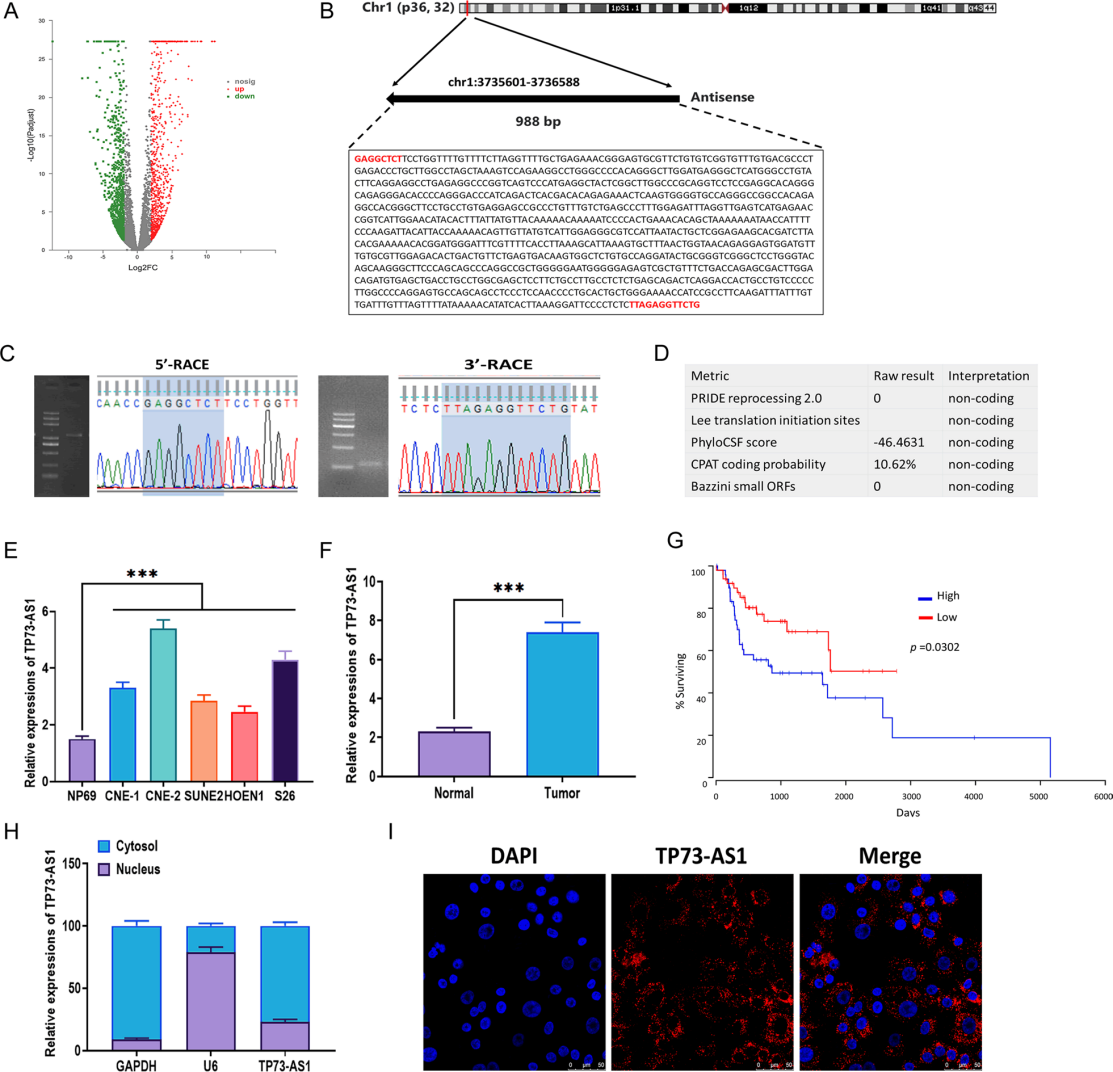
TP73-AS1 is involved in the progression of NPC. **A** Differentially expressed lncRNAs in NPC tissues relative to adjacent healthy tissues. Data were obtained from microarray data, GSE95166 (TCGA). **B** Sequence information and full-length sequence of TP73-AS1. **C** Gel electrophoresis of TP73-AS1 5′- and 3′-RACE PCR products and sequences. **D** Protein coding potential of TP73-AS1, as predicted by 5 metrics. **E** Expression of TP73-AS1 in NPC cell line CNE-1, CNE-2, SUNE2, HOEN1, and S26 compared with normal nasopharyngeal epithelial cell line NP69. **F** Expression of TP73-AS1 in NPC tissues and normal tissues. **G** Overall survival of NPC patients with high or low expression of TP73-AS1. **H** Expression of TP73-AS1 in the subcellular fractions of CNE-2 cells. **I** Subcellular distribution of TP73-AS1, as illustrated by the fluorescence in situ hybridization (FISH) assay (Scalar bar = 50 μm) (***p < 0.001)

Through screening the lncRNA expression profile in the GSE95166 database, we aimed to identify the potential lncRNAs associated with the progression of NPC. Among a group of upregulated lncRNAs in NPC, TP73-AS1 displayed a notable increase in NPC, as shown in the volcano map (Fig. 1A). Based on the sequence information in LNCipedia database [35] and 5′- and 3′-RACE assays, TP73-AS1 was 988 bp in length and was located on chromosome 1 and only contained one exon (Fig. 1B, C). According to the predictions from five metrics, TP73-AS1 rarely possessed the potential of protein-coding (Fig. 1D). Next, we determined the expression of TP73-AS1 in several NPC cell lines, including CNE-1, CNE-2, SUNE2, HOEN1, and S26. Compared with the control nasopharyngeal epithelial cell line NP69, TP73-AS1 expression was notably increased in all NPC cell lines, of which CNE-2 showed the highest upregulation (Fig. 1E). Meanwhile, TP73-AS1 was also upregulated in the NPC tumor tissues relative to normal adjacent tissues (Fig. 1F). Overall, survival analysis revealed that NPC patients with a high level of TP73-AS1 were associated with more unsatisfactory prognostic outcomes (Fig. 1G). For the distribution of TP73-AS1, both subcellular distribution and FISH assays illustrated that TP73-AS1 was primarily located in the cytoplasm, not the nucleus (Fig. 1H, I).

### Effect of TP73-AS1 on the proliferation of NPC cells

**Fig. 2:**
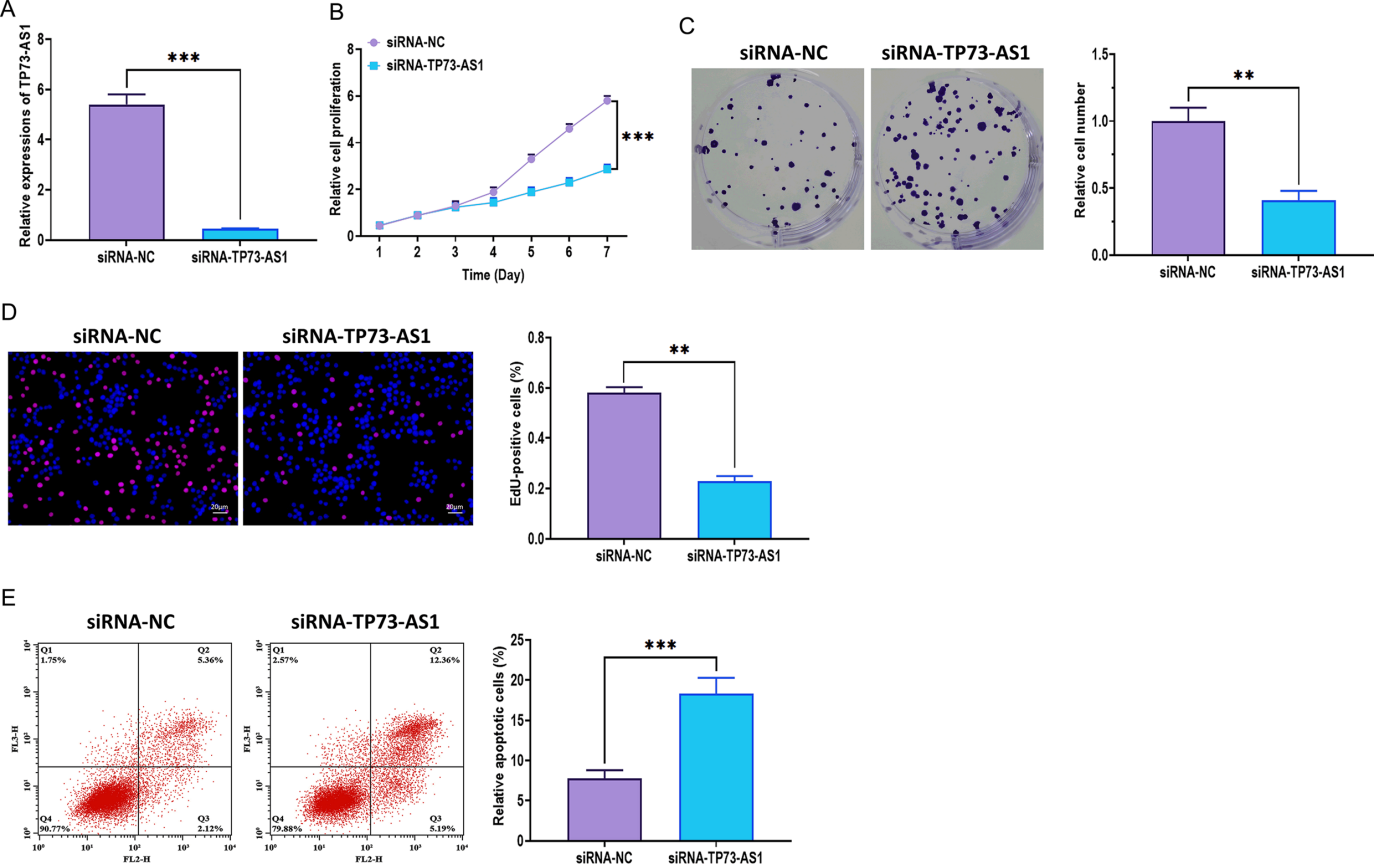
TP73-AS1 knockdown inhibits the proliferation of NPC cells. **A** Knockdown efficacy of siRNAs against TP73-AS1 (siRNA-TP73-AS1). **B** Proliferation of NPC CNE-2 cells with TP73-AS1 knockdown, as illustrated by the MTT assay. **C** Colony formation of NPC CNE-2 cells with TP73-AS1 knockdown. **D** Proliferation of NPC CNE-2 cells with TP73-AS1 knockdown, as illustrated by the EdU assay (Scalar bar = 20 μm). **E** Apoptosis of NPC CNE-2 cells with TP73-AS1 knockdown, as illustrated by the flow cytometry assay (**p < 0.01 and ***p < 0.001)

**Fig. 3:**
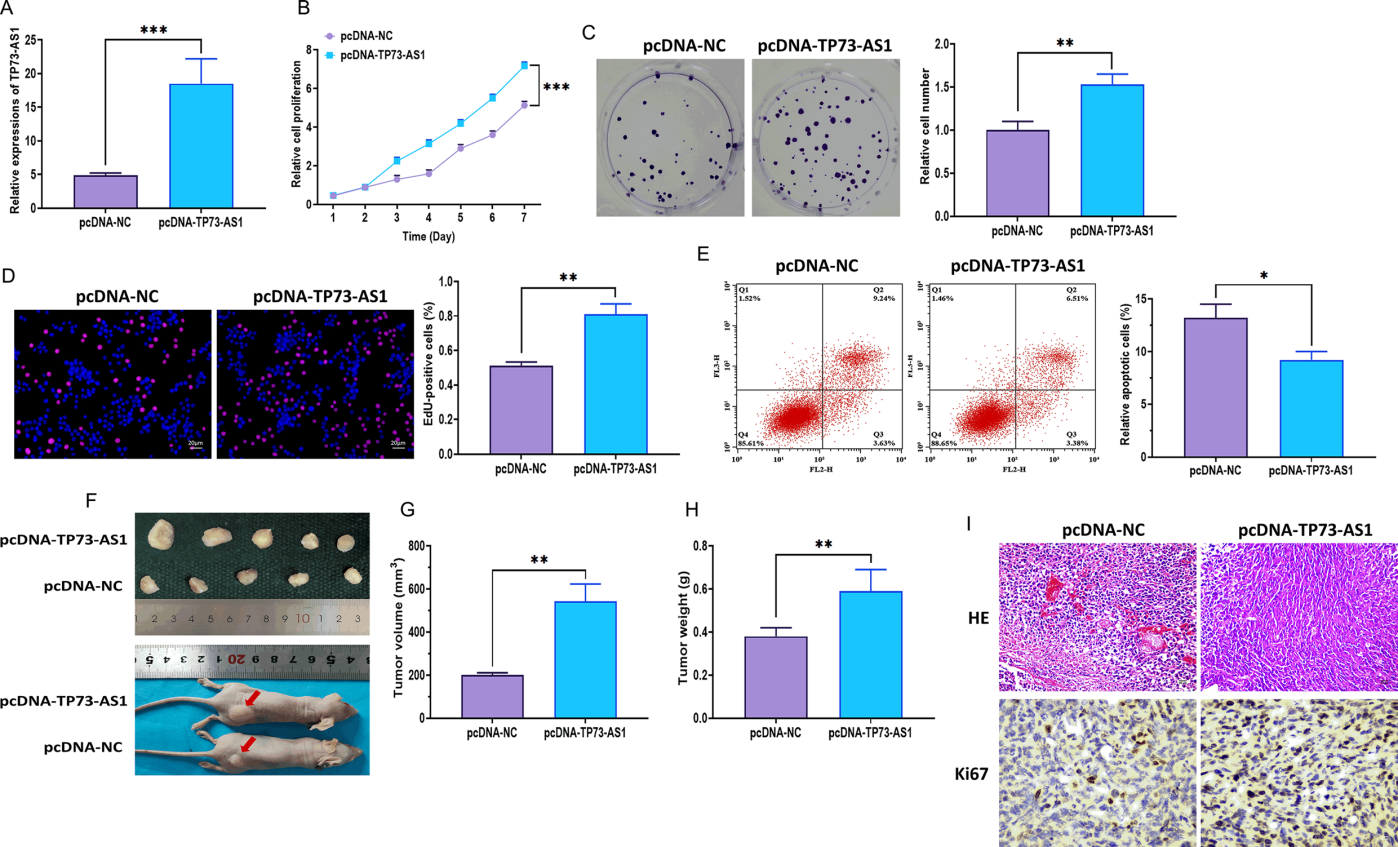
TP73-AS1 overexpression promotes the proliferation of NPC cells in vitro and in vivo. **A** Overexpression efficacy of pcDNA3.1 plasmid carrying TP73-AS1 sequence. **B** Proliferation of NPC CNE-2 cells with TP73-AS1 overexpression, as illustrated by the MTT assay. **C** Colony formation of NPC CNE-2 cells with TP73-AS1 overexpression. **D** Proliferation of NPC CNE-2 cells with TP73-AS1 overexpression, as illustrated by the EdU assay (Scalar bar = 20 μm). **E** Apoptosis of NPC CNE-2 cells with TP73-AS1 overexpression, as illustrated by the flow cytometry assay. **F** Representative images of NPC tissues and xenograft mice. **G** Tumor volume of NPC tissues. **H** Tumor weight of NPC tissues. **I** H&E and Ki67 staining in NPC tissues (Scalar bar = 20 μm) (*p < 0.05; **p < 0.01 and ***p < 0.001)

To elucidate the role of TP73-AS1 in the proliferation of NPC cells, we applied short interference RNAs (siRNA-TP73-AS1) and the pcDNA3.1 plasmid (pcDNA-TP73-AS1) to knockdown and overexpress TP73-AS1 in CNE-2 cells, respectively. The transfection efficacy of siRNAs and pcDNA3.1 were evaluated using quantitative real-time PCR (Figs. 2A and 3A). Next, we found that TP73-AS1 knockdown significantly inhibited proliferation, colony formation, and DNA synthesis (Fig. 2B–D). Also, the downregulation of TP73-AS1 notably promoted apoptosis of CNE-2 cells (Fig. 2E). Oppositely, TP73-AS1 overexpression promoted proliferation, colony formation, and DNA synthesis (Fig. 3B–D) while suppressed apoptosis of CNE-2 cells (Fig. 3E). In addition, the function of TP73-AS1 overexpression in the xenograft mouse model was found to be consistent with in vitro studies. The results showed that forced expression of TP73-AS1 significantly promoted tumor growth in vivo (Fig. 3F–H). Furthermore, IHC assay illustrated that TP73-AS1 overexpression was associated with increased levels of Ki67, suggesting that cell proliferation was enhanced (Fig. 3I). Together, these results demonstrated that TP73-AS1 may be essential for the proliferation of NPC cells in vitro and in vivo.

### TP73-AS1 directly targets miR-342-3p

**Fig. 4:**
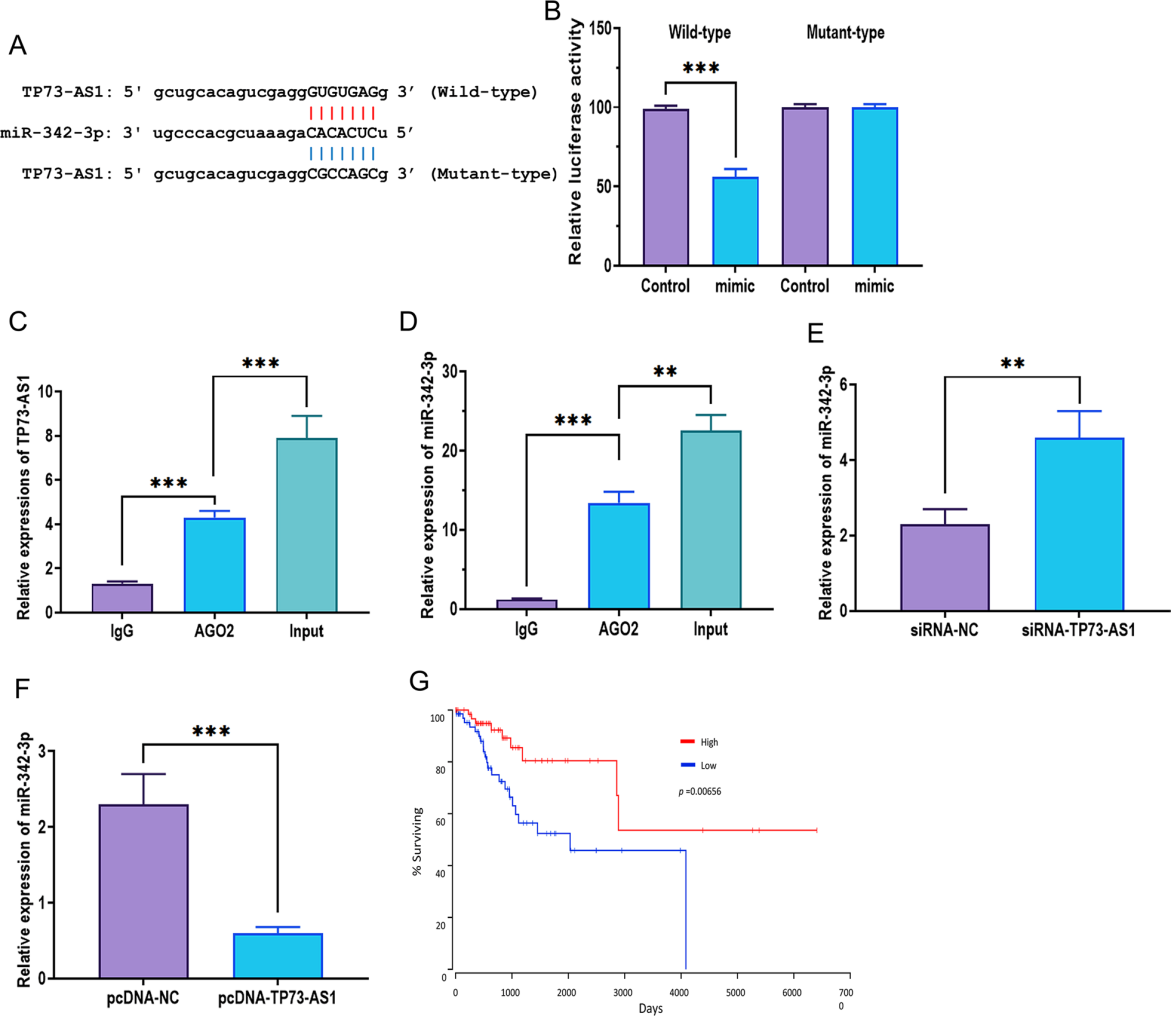
TP73-AS1 directly targets miR-342-3p. **A** Putative binding site of miR-342-3p in TP73-AS1 sequence. **B** Luciferase assay in HEK293T cells transfected with wild- or mutant- type TP73-AS1 and miR-342-3p mimic or control. **C**,**D** Interaction of AGO2 with TP73-AS1 or miR-342-3p, as illustrated by the RIP assay. **E** Expression of miR-342-3p in NPC CNE-2 cells with TP73-AS1 knockdown. **F** Expression of miR-342-3p in NPC CNE-2 cells with TP73-AS1 overexpression. **G** Overall survival of NPC patients with high or low expression of miR-342–3 (**p < 0.01 and ***p < 0.001)

As one of the essential mechanisms underlying their biological functions, lncRNAs act as miRNA sponges to regulate the expression and functions of miRNAs [36, 37]. Through the analysis in MiRanda (http://www.microrna.org) and TargetScan (http://www.targetscan.org), miR-342-3p may be a potential target of TP73-AS1 (Fig. 4A). The luciferase reporter assay revealed that overexpression of miR-342-3p decreased the luciferase activity of HEK293T cells transfected with the wild-type TP73-AS1 vector, but not in those treated with the mutant-type vector (Fig. 4B). The immunoprecipitation assay showed that both TP73-AS1 and miR-342-3p could be pulled down by the AGO2 antibody (Fig. 4C, D), further verifying the physical interaction between TP73-AS1 and miR-342-3p. In addition, TP73-AS1 knockdown upregulated the level of miR-342-3p (Fig. 4E), whereas TP73-AS1 overexpression exerted opposite roles (Fig. 4F). Overall, the survival analysis revealed that patients with a low expression of miR-342-3p displayed worse prognostic outcomes compared with those with high levels of miR-342-3p (Fig. 4G).

### Effect of TP73-AS1 on NPC cells is mediated by miR-342-3p

**Fig. 5:**
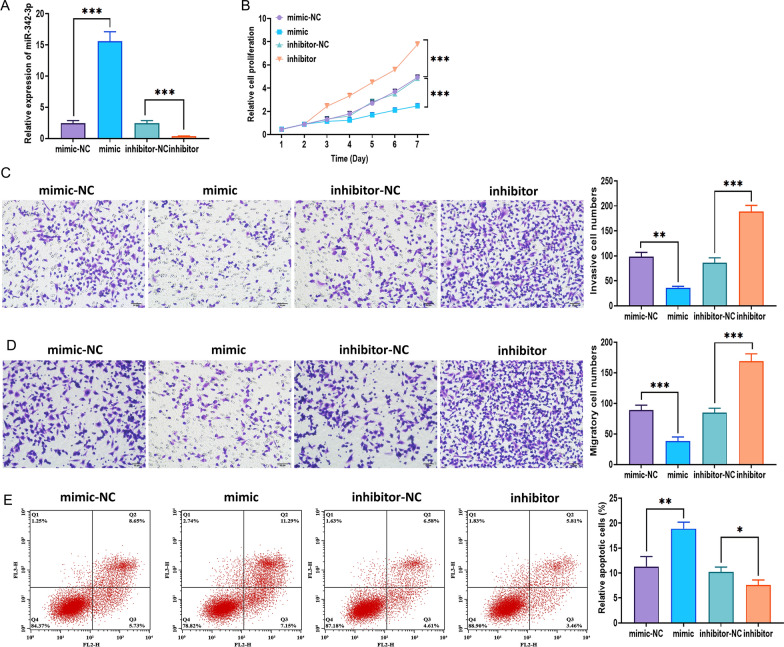
MiR-342-3p affects the progression of NPC CNE-2 cells. **A** Transfection efficacy of miR-342-3p mimic and inhibitor. **B** Proliferation of NPC CNE-2 cells with miR-342-3p overexpression and knockdown, as illustrated by the MTT assay. **C** Apoptosis of NPC CNE-2 cells with miR-342-3p overexpression and knockdown, as illustrated by the flow cytometry assay. **D** Invasion ability of NPC CNE-2 cells with miR-342-3p overexpression and knockdown (Scalar bar = 50 μm). **E** Migration ability of NPC CNE-2 cells with miR-342-3p overexpression and knockdown (Scalar bar = 50 μm) (*p < 0.05; **p < 0.01 and ***p < 0.001)

**Fig. 6:**
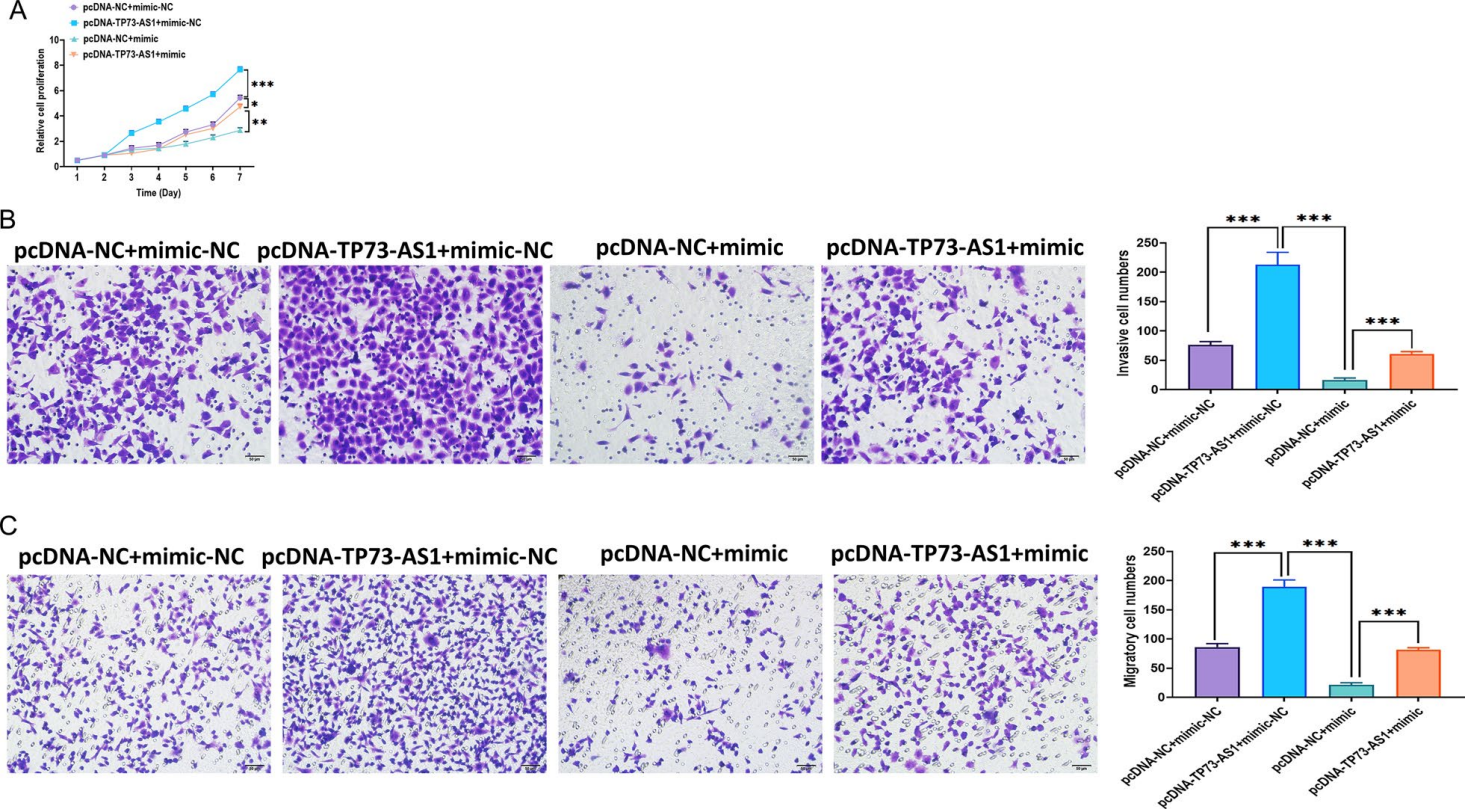
MiR-342-3p attenuates the effect of TP73-AS1 overexpression on NPC CNE-2 cells. **A** Proliferation of NPC CNE-2 cells with TP73-AS1 overexpression, miR-342-3p overexpression, or both, as illustrated by the MTT assay. **B** Invasion ability of NPC CNE-2 cells with TP73-AS1 overexpression, miR-342-3p overexpression, or both (Scalar bar = 50 μm). **C** Migration ability of NPC CNE-2 cells with TP73-AS1 overexpression, miR-342-3p overexpression, or both (Scalar bar = 50 μm) (*p < 0.05; **p < 0.01 and ***p < 0.001)

To investigate the effect of miR-342-3p, we applied the miR-342-3p mimic and inhibitor to overexpress and knockdown the level of miR-342-3p, respectively, and the transfection efficacy was evaluated through quantitative real-time PCR (Fig. 5A). As expected, miR-342-3p overexpression significantly inhibited the proliferation, invasion, and migration, while promoted apoptosis of CNE-2 cells (Fig. 5B–E). On the other hand, miR-342-3p knockdown displayed the opposite roles (Fig. 5B–E). Furthermore, we conducted rescue experiments to further determine the functions of TP73-AS1 and miR-342-3p in NPC cells. The results demonstrated that the effect of TP73-AS1 overexpression on the proliferation, invasion, and migration of CNE-2 cells was attenuated by forced expression of miR-342-3p (Fig. 6A–C), indicating the essential role of the TP73-AS1/miR-342-3p pathway in NPC cells.

### TP73-AS1 is transferred by exosomes to promote M2 polarization

**Fig. 7:**
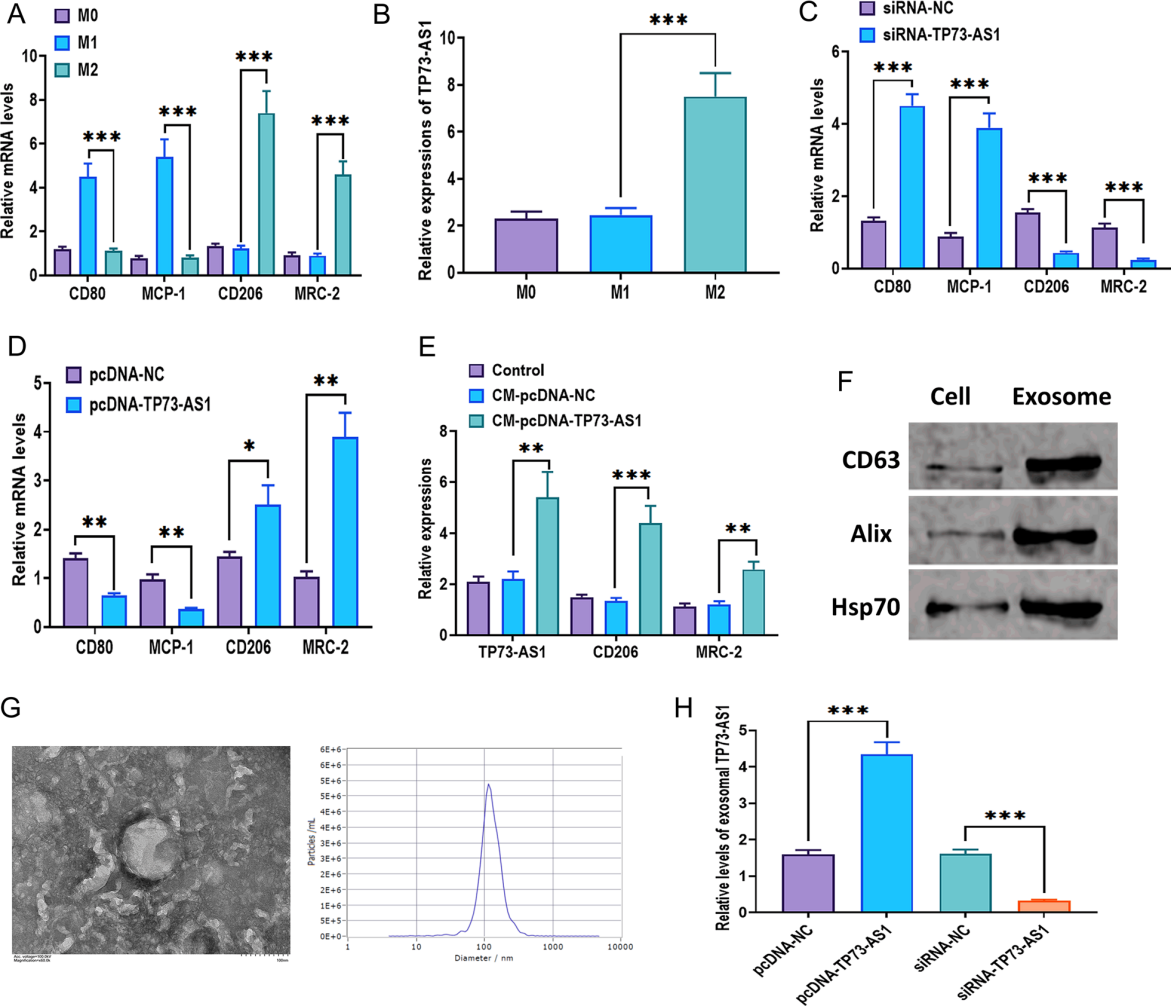
TP73-AS1 is transferred by exosomes to promote M2 polarization. **A** mRNA expressions of M1 and M2 markers in macrophages treated with LPS/INF-γ or IL-4/IL-13. **B** Expression of TP73-AS1 in M0, M1, and M2 macrophages. **C** mRNA expressions of M1 and M2 markers in macrophages with TP73-AS1 knockdown. **D** mRNA expressions of M1 and M2 markers in macrophages with TP73-AS1 overexpression. **E** Expressions of TP73-AS1 and M2 markers in macrophages treated with conditioned medium of TP73-AS1-overexpression CNE-2 cells. **F** Expressions of exosomal markers in exosomes derived from CNE-2 cells, as illustrated by western blotting. **G** Morphology and size distribution of exosomes derived from CNE-2 cells, as illustrated by transmission electron microscopy and nanoparticle tracking analysis (Scalar bar = 100 μm). **H** Level of exosomal TP73-AS1 derived from CNE-2 cells with TP73-AS1 overexpression or knockdown (*p < 0.05; **p < 0.01 and ***p < 0.001)

It has been demonstrated that tumor-associated macrophages, especially the M2 phenotype, play a critical role in the progression and development of cancers [38, 39]. To determine the status of macrophages, we measured the expressions of M1 (CD80 and MCP-1) and M2 (CD206 and MRC-2) markers in control macrophages, M1 macrophages, and M2 macrophages (Fig. 7A). The level of TP73-AS1 was significantly increased in M2 macrophages (Fig. 7B). After TP73-AS1 knockdown, expressions of M1 markers, CD80 and MCP-1, were increased, and levels of M2 markers, CD206 and MRC-2, were decreased (Fig. 7C). The opposite changing trends were observed in cells with TP73-AS1 overexpression (Fig. 7D). Intriguingly, the conditioned medium cultured by TP73-AS1-overexpressing CNE-2 cells increased the levels of M2 markers relative to CNE-2 cells-conditioned medium (Fig. 7E). In addition, exosomal lncRNAs are essential for intercellular communication in the TME [40, 41]. To further elucidate the mechanism underlying the interaction between NPC cells and macrophages, we speculated that TP73-AS1 may be released from NPC cells via exosomes. Through the hyper-centrifugation assay, we isolated exosomes from the conditioned medium cultured TP73-AS1-overexpressing CNE-2 cells. The protein expressions of exosomal markers CD63, Alix, and Hsp70 were increased in exosomes compared with CNE-2 cells (Fig. 7F). Transmission electron microscopy and nanoparticle tracking analysis were also performed to validate the morphology and size distribution of exosomes. The results showed that exosomes displayed a round-shape with cup-like concavity and 80–140 nm in diameter (Fig. 7G). Moreover, TP73-AS1 overexpression and knockdown in CNE-2 cells upregulated and downregulated the level of exosomal TP73-AS1, respectively (Fig. 7H).

### Exosomal TP73-AS1 promotes the motility and tube formation of macrophages

**Fig. 8:**
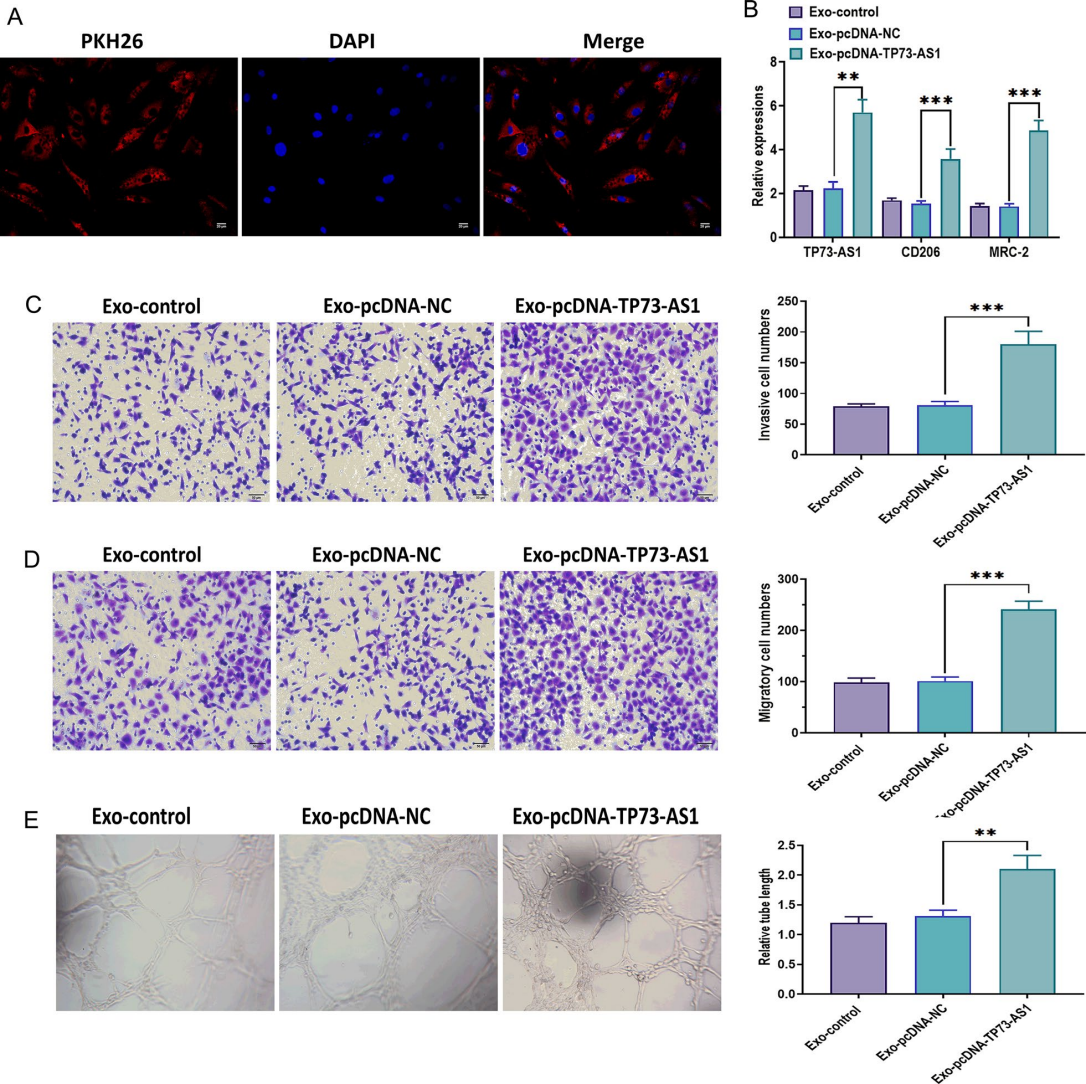
Exosomal TP73-AS1 promotes the motility and tube formation of macrophages. **A** Exosomal TP73-AS1 was taken up by macrophages, as illustrated by the fluorescence in situ hybridization (FISH) assay (Scalar bar = 50 μm). **B** Expressions of TP73-AS1 and M2 markers in macrophages treated with exosomes derived from TP73-AS1-overexpressing CNE-2 cells. **C** Invasion ability of macrophages treated with exosomes derived from TP73-AS1-overexpressing CNE-2 cells (Scalar bar = 50 μm). **D** Migration ability of macrophages treated with exosomes derived from TP73-AS1-overexpressing CNE-2 cells (Scalar bar = 50 μm) (E) Ability of tube formation of macrophages treated with exosomes derived from TP73-AS1-overexpressing CNE-2 cells (**p < 0.01 and ***p < 0.001)

To elucidate the role of exosomal TP73-AS1 in macrophages, we cocultured macrophages with exosomes derived from TP73-AS1-overexpressing CNE-2 cells. FISH assays revealed that red PKH26-labeled exosomes were taken up by macrophages (Fig. 8A). The expressions of TP73-AS1, CD206, and MRC-2 were increased in macrophages after treating with exosomes (Fig. 8B). Additionally, exosomes derived from TP73-AS1-overexpressing CNE-2 cells significantly promoted invasion, migration, and tube formation of macrophages (Fig. 8C–E). Collectively, these results suggest that exosomal TP73-AS1 could be transferred from NPC cells to macrophages, thereby regulating M2 polarization and the behaviors of macrophages.

## Discussion

Recently, lncRNAs have been widely recognized as essential regulators for tumorigenesis and cancer progression [42, 43]. Meanwhile, with advances in bioinformatics and high-throughput genome analysis techniques, increasing valuable public databases are available for investigating the diagnosis, treatment, and prevention of cancers, such as TCGA [44]. In the present study, we analyzed microarray data (GSE95166) in the TCGA dataset and found that TP73-AS1 was overexpressed in NPC tissues and cell lines. We also observed that TP73-AS1 overexpression could significantly promote the progression of NPC, whereas TP73-AS1 knockdown resulted in the opposite effect. Furthermore, TP73-AS1 was associated with tumor growth of xenograft mice in vivo. As a multifunctional factor, TP73-AS1 has been demonstrated to be involved in various cancers. For example, high levels of TP73-AS1 is related to poor prognosis in glioblastoma and enhances temozolomide resistance in cancer stem cells [45]. In ovarian cancer, the oncogenic effect of TP73-AS1 is associated with cell proliferation and metastasis through the matrix metallopeptidase 2/9 pathway [46]. On the other hand, the anti-tumor role of TP73-AS1 has been reported in bladder cancer, in which TP73-AS1 overexpression suppresses cell migration, invasion, and metastasis through inactivating epithelial-mesenchymal transition (EMT) [47]. As such, the functions of TP73-AS1 in cancers display cancer-type-dependent manners, indicating that TP73-AS1 is involved in multiple regulatory signaling pathways to regulate cancer progression and TP73-AS1 may be a potential target for treating cancers.

Given the observations mentioned above, we aimed to further elucidate the molecular mechanism underlying the role of TP73-AS1 in NPC. In cancers, lncRNAs have been demonstrated to regulate the expression of miRNAs through various mechanisms. One well-studied mechanism includes that of lncRNAs function as endogenous miRNA sponges to keep miRNAs from biding with target genes [48]. In the present study, bioinformatics analysis and mechanical experiments collectively demonstrated that TP73-AS1 could directly target miR-342-3p, and there was a negative correlation between TP73-AS1 and miR-342-3p. Functionally, the effect of TP73-AS1 on NPC was attenuated by the overexpression of miR-342-3p. These findings together suggest that TP73-AS1 acts as a competing endogenous RNA (ceRNA) by sponging miR-342-3p, thereby promoting NPC progression. Similarly, miR-342-3p can also be sponged by other lncRNAs, thereby exerting an anti-tumor role in other cancer types, including pancreatic cancer [49], gallbladder cancer [50], and gastric cancer [51]. Therefore, the interaction between lncRNAs and miRNAs could provide a new avenue for cancer treatment.

TME is a complicated micro-ecosystem of tumor cells and provides supportive external context for the proliferation of tumor cells during carcinoma tumorigenesis and development [52]. A growing body of research demonstrated that macrophages, a class of adaptive and innate immune cells, are highly abundant in the entire process of tumor progression [53]. Over the past decade, the mechanism of intercellular communication between tumor cells and macrophages of TME has emerged as a hot topic in cancer research [18, 54, 55]. Among previous findings, exosomes, essential information transporters in intercellular communication, are regarded as one of the critical mechanisms for the interaction between tumor cells and their TME [56]. In the present study, we demonstrated that exosomes derived from NPC cells could transport TP73-AS1 to TAMs, thereby promoting the motility and tube formation of TAMs. Similar observations are also reported in other cancers. For example, Hsieh et al. reported that exosomal miR-21 derived from head and neck cancer cells facilitates the polarization of TAMs [56]. In addition, Gerloff et al. found that exosomes derived from cutaneous melanoma cells induce tumor-promoting phenotypes of TAMs through transporting miR-125b-5p [57]. Therefore, exosome-based intercellular communication may serve as an essential promotor for tumor progression and provide a potential strategy for intercepting TAMs-associated TME remodeling.

To the best of our knowledge, this is the first report regarding the function of exosomes derived from NPC cells in TAMs in NPC. Meanwhile, this study demonstrated that TP73-AS1 could be transported from NPC cells to TAMs, thus, influencing the cellular activities of TAMs. However, there are still some limitations that should be further addressed in future studies. First, the function of NPC-derived exosomes should be studied in the xenograft mouse model. Second, given the multifunctional properties of TP73-AS1 and miR-342-3p, the downstream target of either TP73-AS1 or miR-342-3p should be further explored. Lastly, more clinical specimens and patient’s information should be included to determine the diagnostic value of TP73-AS1 in NPC.

## Conclusion

In summary, the results demonstrated that TP73-AS1 acts as an oncogenic factor in NPC through sponging miR-342-3p. Also, exosomal TP73-AS1 derived from NPC cells promotes M2 polarization of macrophages. Our findings provide a new understanding of the molecular mechanism of NPC and an emerging therapeutic strategy for the treatment of NPC.

## Data Availability

The datasets used and/or analyzed during the current study are available from the corresponding author on reasonable request.

## Abbreviations

NPC: Nasopharyngeal carcinoma
lncRNAs: Long noncoding RNAs
TAMs: Tumor-associated macrophages
TME: Tumor microenvironment
TCGA: The Cancer Genome Atlas
RPMI: Roswell Park Memorial Institute
FBS: Fetal bovine serum
HEK293T: Human embryonic kidney 293 cells
DMEM: Dulbecco’s modified Eagle’s medium
FISH: Fluorescence in situ hybridization
H&E: Hematoxylin and eosin
RIP: RNA immunoprecipitation
NTA: Nanoparticle tracking analysis
EMT: Epithelial–mesenchymal transition
ceRNA: Competing endogenous RNA
